# Evaluation of a novel high speed burr tip for safe and efficient bone surgery in a sheep model

**DOI:** 10.1038/s41598-025-01769-7

**Published:** 2025-05-15

**Authors:** Nuutti Vartiainen, Mika Niemelä, Esa-Pekka Pälvimäki

**Affiliations:** 1 Department of Neurosurgery, Helsinki University Hospital, University of Helsinki, Helsinki, Finland; 2 Department of Neurosurgery, Helsinki University Hospital, Haartmaninkatu 4, Helsinki, 00029 Finland

**Keywords:** Burr, Dural tear, High-speed drill, New technology, Soft tissue injury, Surgical instrument, Neurology, Engineering

## Abstract

The exposure of neural structures by removing the overlying bone using a high-speed drill can cause iatrogenic injury to the dura, neural tissue, and vessels. We investigated the safety and efficacy of a novel Surgify Safety Burr (SSB), attachable to existing high-speed drills. Three anesthetized living sheep underwent cranial burr hole drilling (*n* = 19) and hemilaminectomies (*n* = 14) with the SSB and standard burrs. Drilling time, dural injury, and subjective evaluation of heating and haptic feedback of each burr were assessed. Cranial burr hole drilling was done using diamond (*n* = 3), fluted (*n* = 6), and SSB (*n* = 10) burrs. Penetrating dura damage was observed in 33% of the diamond burr drilled holes, and in 33% of the fluted burr drilled holes, but not in the holes drilled with the SSB. Hemilaminectomies were performed with the SSB (*n* = 7) and diamond (*n* = 7) burrs without drilling-induced dura damage. Mean drilling time was not significantly different in cranial drilling (SSB, 121 ± 18s; fluted burr, 63 ± 22s; diamond burr, 165 ± 29s) nor in hemilaminectomy (SSB, 108 ± 13s; diamond burr, 95 ± 18s). Subjective evaluation of the SSB suggested efficient bone removal without heating or chattering. The SSB is a safe and effective surgical tool in cranial drilling and hemilaminectomy procedures. Safety, efficacy, and clinical benefits should be established in human studies.

## Introduction

High-speed drills and their burr attachments are essential tools in modern surgery. In neurosurgery, the fundamental surgical step is the bony exposure of the neural structures to be operated upon. This exposure, the surgical opening, is typically carried out with a craniotome and high-speed drills. Bone drilling carries the inherent risk of unintended injuries by direct contact between the burr tip and surrounding structures such as the dura, nervous tissue, blood vessels, or by indirect heating damage. In spinal surgery, the incidence of unintended durotomy varies between 1.6 and 12.4%^1–8^, and can vary with the complexity of the surgery performed^5^. Incidental durotomy has been associated with longer hospital stays, increased complications and postoperative morbidity, and increased hospital costs^3,9,10^. Heating of the drill is also a common phenomenon, and indirect thermal damage of nerve roots and bone has been previously described^11,12^.

Ultrasonic technologies offer an alternative means to remove bone. This technique has been used since the 1950s in dental and maxillofacial surgery, and recently also in neurosurgery. Ultrasonic bone cutting can be used for various spinal operations such as decompression and laminoplasty^13–15^. However, this technique showed a comparable safety profile to conventional decompression techniques with no difference in the number of iatrogenic dural tears^10,16^. Studies have shown generation of high heat^17,18^ and dural and spinal cord injuries^19^ with ultrasonic bone cutting. Moreover, although laminectomy performed with a pediatric craniotome drill with a footplate attachment was reported to cause less iatrogenic dural tears than high-speed drilling, such complications are still observed^8^. Therefore, a safe and effective technology to surgically remove bone is currently lacking.

**Fig. 1:**
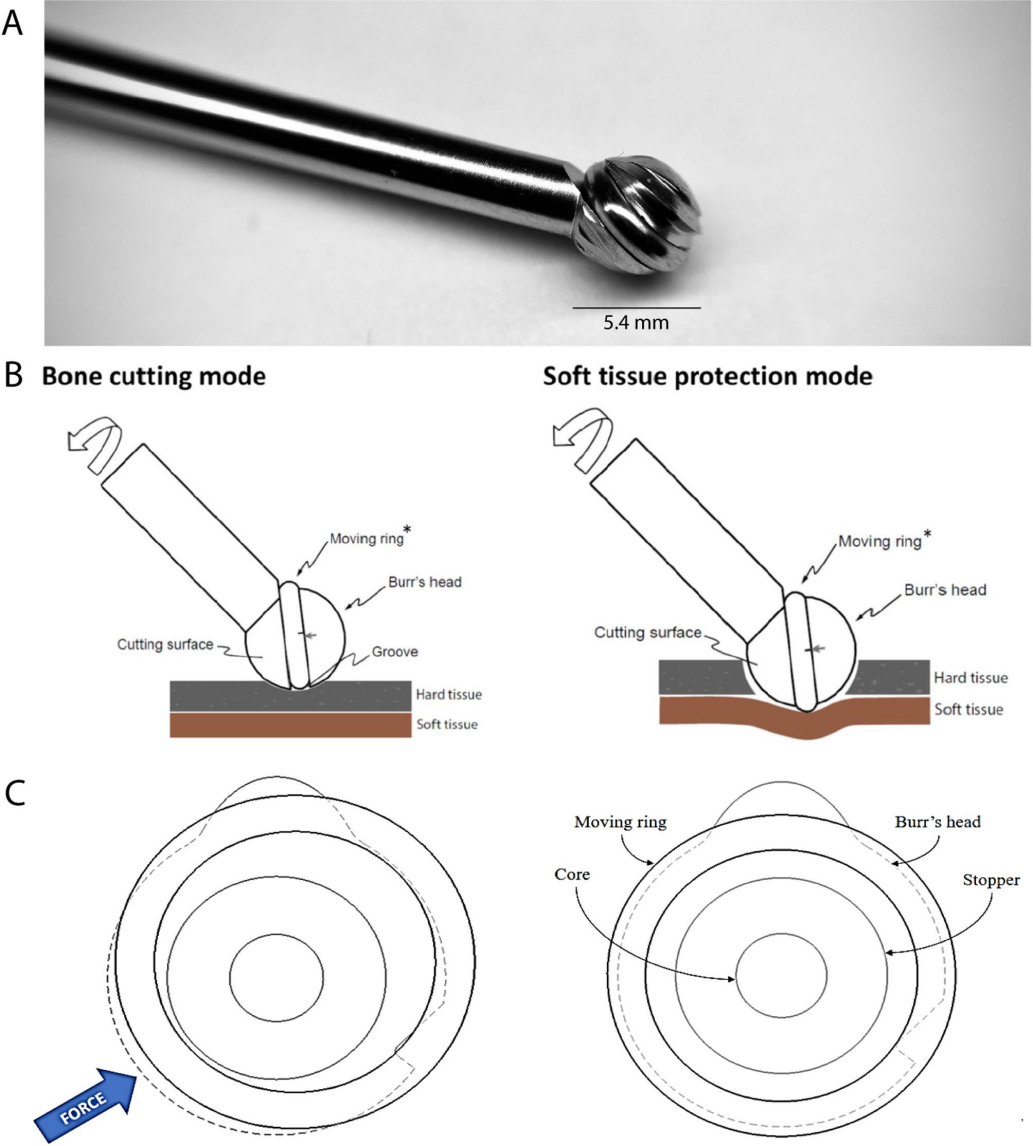
(**A**) The Surgify Safety Burr (SSB), including a (**B**) description showing the parts and differential modes in bone cutting and soft tissue protection mode, and (**C**) a detailed scheme of the different positions of the moving ring where the tool is not in contact with hard tissue/bone (left), and when it is pressed against soft tissue (right). The length of the burr shaft is 85 mm (medium version) and 140 mm (long version) and the diameter of the burr head is 5.4 mm.

To reduce the risk of direct mechanical damage and indirect thermal damage from a high-speed drill, the Surgify Safety Burr (SSB), a novel CE-marked medical device approved for clinical use in the EU and in the US has been developed (Fig. 1A). The SSB is a tissue-selective burr for neurosurgical, spinal, ear, and general surgical procedures, and can be attached to existing high-speed drills. The device’s safety mechanism consists of a dynamic shield around the burr head that neutralizes the cutting edges whenever the device is not in contact with hard tissue. Moreover, it provides higher levels of control and precision through reduced chattering (sudden, uncontrollable, and dangerous jumping of the burr) compared to conventional burrs, ensuring accuracy and stability in bone removal procedures. Additionally, the SSB cutting speed reduces automatically when a thin layer of bone starts to form, giving extra time for the surgeon to react before reaching the soft tissue underneath the bone^20^.

For the first time, here we investigated the use of this burr tip to drill cranial and spinal bone of living sheep, as to mimic typical neurosurgical cranial and spinal procedures in humans. Our aim was to study the safety and efficacy of the SSB in living sheep and compare it to conventional diamond and fluted burrs. The parameters evaluated were the occurrence of dura lesions, drilling and procedure times, as well as subjective evaluation of heating and chattering.

## Methods and materials

### SSB technical description

The SSB is made of medical-grade metals and is compatible with existing high-speed drill systems (Fig. 1A) and can be purchased from Surgify Medical Oy (reference numbers 100-54-TM and 100-54-TL)^21^. The SSB comprises of (1) a shank that transfers rotary movement of the drill motor to the head, (2) a locking feature at the distal end of the shank to allow the burr shank to be locked into a high-speed surgical drill, (3) a head part that is shaped with two bone-cutting edges, and (4) a safety mechanism consisting of a moving ring and an elastic component installed in the burr head, used to return the ring to the center position. The ring has a larger outer diameter than the burr head and can have limited radial movement inside a groove around the burr head. This ring acts as a safety shield and can be deactivated only when the burr head is pushed against a hard material such as bone. Depending on the pressure applied and the resistance it encounters, the moving ring can gradually move between two different positions. In the bone cutting mode, the centered ring retracts into a groove within the burr to a level lower than the cutting edges of the burr to allow the burr to cut the targeted tissue. In the soft tissue protection mode, the off-centered ring protrudes to a level higher than the cutting edges and prevents the burr edges from engaging with and cutting soft tissue^22,23^. This feature significantly reduces damage to the sensitive soft tissues (Fig. 1B,C). The length of the SSB is 85 mm (medium version for cranial drilling, reference number 100-54-TM) and 140 mm (long version for spinal drilling, reference number 100-54-TL) and the diameter of the burr head is 5.4 mm. Figure 1 presents the SSB and its underlying safety mechanism.

### Setting and test animals

The study was conducted using three sheep (ewes, aged 2–4 years). No specific criteria were set for the inclusion or exclusion of animals. The sheep were acquired from domestic sheep farms through the Large Animal Center of the University of Helsinki (Viikki, Helsinki, Finland) and were kept at the same facility with an acclimatization period of at least two weeks. The Large Animal Center of the University of Helsinki complies with the legal requirements regarding the maintenance of the animals. The sheep were given preoperative sedation with medetomidine (10–40 μg/kg, IM). Propofol (2–4 mg/kg, IV) was used for induction of the anesthesia and isoflurane (1–2.5%) for maintenance. The depth of the anesthesia was monitored with a pulse oximeter and by monitoring breathing rate and pain reflexes. Buprenorphine (0.01 mg/kg, IV) was administered intraoperatively as pain medication. After the procedures (see Surgical methods), sheep were sacrificed immediately without resuscitation with an overdose of pentobarbital. Reporting of this study was done according to the Animal Research: Reporting of In Vivo Experiments (ARRIVE) reporting guidelines^24^.

The anatomy of the sheep is favourable for studying surgical drills both in spinal and cranial applications, due to the accessibility of the spine and the relatively round calvaria. In order to simulate the status on a living patient, the procedures needed to be carried out on a living anesthetized animal, as only then the pressure of the cerebrospinal fluid pushes the soft tissues against the bony structures around the central nervous system. The number of animals was as small as possible to achieve sufficient reliability of the results giving the experimental nature of the study. In addition, animals underwent several craniotomy procedures on the head area, as well as laminectomy procedures in the back area during the same operation to further minimize the number of animals needed.

### Surgical methods

The head of the sheep was immobilized with a Sugita frame. A cranial skin incision was performed at the midline. Standard retractors were used to expose the sheep skull. Burr holes were drilled into the skull with SSB, a diamond drill and a fluted burr. The size of the drill tip was 5–5.5 mm. The drill console and motor were manufactured by Stryker. The SSB was operated at 37 000 rpm, and the diamond drill and fluted burrs were operated at 75 000 rpm. The order of the burr holes and the burr tip (SSB, diamond, fluted) was pseudorandomized. Any possible remaining thin layer of bone was removed with a Kerrison Rongeur (punch). Investigators could not be blinded to the burr tip type as the drilling was performed under visual control and the type of the tip was evident to the user.

Hemilaminectomies were performed with SSB and diamond burr (coarse and standard). Standard microdecompression and hemilaminectomy surgical methods were used. Briefly, a midline skin incision, separation of muscle from spinous process and lamina, and application of a Caspar retractor were done, followed by microscopic (high magnification) decompression consisting of medial facet thinning by drilling, and finally hemilaminectomy via drilling and Kerrison punch. Flavectomy was then performed and further exposure of the dural sac to emulate decompression of the midline to lateral dural sac and recess was done. Drilling and a Kerrison punch were used as needed to finalize exposure. Drilling was performed with one hand and burr rotation speed was pedal-controlled by the surgeon according to the surgeon’s preference. For each cranial burr hole and each hemilaminectomy, one burr was used.

### Outcome measures

For each drilling experiment, the presence of dura lesions, the drilling time, and procedure time were measured. Dura lesion was defined as an accidental puncture or laceration of dura during a procedure. The drilling time accounts only for the time when the burr was actively working on the bone (touching the bone), whereas the procedure time was calculated from initiation of drilling until completion of the burr hole/hemilaminectomy.

Heating was evaluated subjectively by the surgeon by somatosensory experience of feeling the heating of the hand piece in the surgeon’s hand, visually by observing the dark, burned discoloration of the bone, and by visual and olfactory observation of smoke formation. Chattering was defined as a phenomenon where the drill bit experiences uncontrolled, rapid vibrations while cutting bone, leading to inefficient and potentially harmful oscillations during surgical procedures.

### Statistical methods

Descriptive analyses were performed to assess the presence of dura lesions, the drilling time, and procedure time. Mean with standard error of mean (SEM) was described for continuous variables. Student’s two-sample unequal variance t-test was used to determine the difference between burrs. Frequencies and proportions were used for categorical variables. Fisher’s exact test was used to determine the occurrence of dura damage with SSB versus conventional burrs.

## Results

### Cranial high-speed bone drilling

**Fig. 2:**
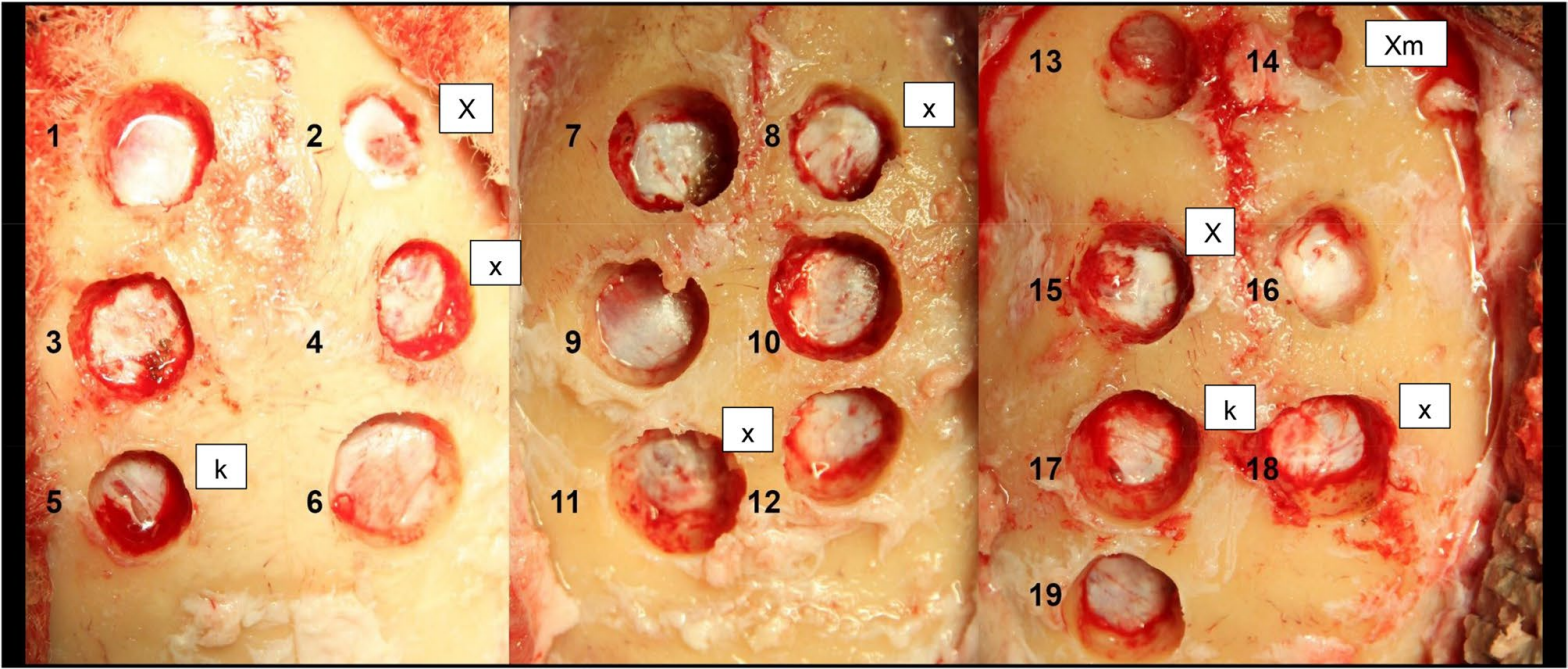
Cranial burr holes from sheep. Tears are referred by the following symbols: X: penetrating dura lesion with high-speed burr; X_m_: penetrating mucosa lesion with high speed burr; x: superficial dura lesion with high speed burr; k: dura lesion with Kerrison punch. Holes were drilled with (**a**) the SSB (1, 3, 6, 7, 10, 12, 13, 16, 17, and 19); (**b**) the fluted burr (4, 9, 11, 14, 15, and 18); and (**c**) the diamond burr (2, 5, and 8).

Altogether, 19 cranial burr holes were done in the three living anesthetized sheep. Six burr holes were made in the first and second sheep, and seven burr holes in the third sheep (Fig. 2). Of the 19 burr holes, ten were made with SSB, six holes with a fluted burr, and three holes with a diamond burr (Table 1).

**Table 1:** Cranial burr hole drilling and procedure times.

Procedure #	Burr	Drilling time (s)	Procedure time (s)	Dura lesion
Sheep 1				
1	SSB™	220	900	
2	Diamond	124	640	X
3	SSB™	147	600	
4	Fluted	48	360	x
5	Diamond	150	420	k
6	SSB™	162	360	
Sheep 2				
7	SSB™	160	600	
8	Diamond	222	540	x
9	Fluted	40	240	
10	SSB™	69	185	
11	Fluted	87	258	x
12	SSB™	80	239	
Sheep 3				
13	SSB™	35	45	
14	Fluted	6	6	X_m_
15	Fluted	35	255	X
16	SSB™	90	220	
17	SSB™	168	376	k
18	Fluted	160	440	x
19	SSB™	79	195	

*X* penetrating dura lesion with high-speed burr, *X*_m_ penetrating mucosa lesion with high-speed burr, *x* superficial dura lesion with high speed burr, *k* dura lesion with Kerrison punch, *SSB* safety burr.

The drilling and procedure times, as well as possible dural injury for each individual burr hole are represented in Table 1. The mean (± SEM) drilling time was 121 ± 18 s for the SSB, 63 ± 22 s for the fluted burr, and 165 ± 29 s for the diamond burr. The mean procedure time was 372 ± 81 s for the SSB, 260 ± 60 s for the fluted burr, and 480 ± 35 s for the diamond burr. Drilling time and procedure time with the SSB did not differ significantly from the other burrs (Table 2).

**Table 2:** Cranial average burr hole drilling and procedure times.

Burr	SSB™	Diamond	*p*-value (SSB™ vs. Diamond)	Fluted	*p*-value (SSB™ vs. Fluted)
Procedure #	10	3	NA	6	NA
Dura lesion	0	2	NA	4	NA
Mean (± SEM) drill time (s)	121 ± 18	165 ± 29	0.273	63 ± 22	0.067
Mean (± SEM) procedure time (s)	372 ± 81	480 ± 35	0.248	260 ± 60	0.286

*SEM* standard error of the mean.

Penetrating dura or mucosa lesions were not experienced when drilling the cranial burr holes with the SSB, whereas these lesions occurred in 33% of the holes produced when using both the fluted (2/6 holes) and the diamond burrs (1/3 holes). Additionally, erosion of superficial dura was observed in one of three burr holes undertaken with the diamond burr (33%) and in two of six burr holes with the fluted burr (33%). Damage of the dura (penetrating or superficial) was observed statistically significantly more often with the conventional burrs (diamond or fluted) than with SSB (Fisher’s exact test, *p* = 0.003). Two dura lesions were inflicted while removing the thin bone rim with the Kerrison punch (Fig. 2, burr holes 5 and 17).

### Hemilaminectomy

Table 3 shows the drilling and procedure times for the 14 hemilaminectomies performed on two living anesthetized sheep. We experienced no dura lesions caused by any of the high-speed drilling burr tips, except for one dura lesion inflicted by the Kerrison punch. The mean drilling time was 108 ± 13 s for the SSB and 95 ± 18 s for the diamond burrs, with the mean procedure time 249 ± 26 s for the SSB and 319 ± 74 s for the diamond burrs (Table 4). Drilling and procedure times with SSB were not significantly different from the diamond burrs.

**Table 3:** Hemilaminectomy drilling and procedure times.

Procedure #	Target	Burr	Drilling time (s)	Procedure time (s)
Sheep 1				
1	L4-5 right	Diamond	35	742
2	L3-4 right	SSB™	77	300
3	L2-3 right	Diamond	184	285
4	L1-2 right^1^	SSB™	170	330
5	T12-L1	Diamond*	116	295
6	T11-T12	SSB™	132	240
Sheep 2				
7	L4-5 right	SSB™	94	321
8		Diamond*	87	263
9	C2 (appr.)	Diamond*	65	138
10	C3	SSB™	75	173
11	L3-4 left	Diamond*	85	220
12	L2-3 left	SSB™	99	170
13	L1-2 left	Diamond*	94	289
14	T12-L1 left	SSB™	108	211

Diamond* = coarse diamond.

^1^ Dura lesion inflicted by the Kerrison punch.

**Table 4:** Average hemilaminectomy drilling and procedure times.

Burr	SSB™	Diamond	*p*-value
Procedure #	7	7	NA
Mean (± SEM) drill time (s)	108 ± 13	95 ± 18	0.570
Mean (± SEM) procedure time (s)	249 ± 26	319 ± 74	0.400

Diamond* = coarse diamond.

*SEM* standard error of the mean, *SSB* safety burr.

#### Subjective evaluation

Subjective observations suggested less heating, discoloration of the drilled bone, and smoke formation with SSB than with the diamond burr. With the SSB, the bone removal appeared faster and less force was needed than with the diamond burr. The SSB appeared to be easier to control manually than the standard fluted burr, and with less chattering.

## Discussion

This is the first study demonstrating the safety and efficacy of SSB in routine neurosurgical procedures. In both cranial burr hole drilling and laminectomy procedures performed on living adult sheep, no damage was observed to the underlying dura compared to the standard fluted and diamond burr tips. In cranial burr hole drilling, we experienced statistically significantly more dura lesions with conventional burrs (diamond and fluted tip) than with the SSB. Although the drilling time did not differ significantly between the SSB and the diamond burr, subjective evaluation suggested that the SSB removes bone faster and with less force. And importantly, the heating effect, normally an evident phenomenon in diamond drilling, was negligible with SSB.

Bone drilling is usually an essential part of neurosurgical procedures, but it is also commonly used in other surgical specialties. Therefore selecting an appropriate burr type based on the surgical context is crucial to minimize complications. Several studies have demonstrated that dura lesions are associated with wound complications, longer hospital stays, and increased health care costs^3,9^. Moreover, it is well described that diamond and ultrasonic bone curette burrs, while providing precision, can generate more heat during bone cutting, potentially leading to thermal damage such as osteonecrosis and coagulative necrosis-like changes in the dura mater^11,12,25^. Therefore, all means that reduce the risk for iatrogenic lesions could lead to increase in the quality of life of these patients and economic savings. The core innovation of the SSB device is the protective mechanism in the tip of the burr that automatically covers the cutting edges when it comes in contact with structures softer than bone. Conversely, the protective mechanism is deactivated when the head is pushed against a hard material such as bone. As the SSB is a replacement for traditional disposable burr tips, it was designed to be used with pre-existing drilling systems.

The results of this study indicated that while the delicate sheep dura was quite frequently penetrated or damaged by the conventional burr tips, no dural damage was seen with the use of SSB. This suggests the clinically relevant safety characteristic of SSB and indicates that it performs as expected according to its intended design. It is noteworthy that even the very delicate mucosa of the sheep frontal sinus remained intact, while the overlying bone was drilled away with the SSB (Fig. 2, see burr hole 13). Such observations suggest a clear advantage of using this device instead of the conventional fluted and diamond burrs in surgical bone drilling. Elia et al. report iatrogenic dural tears in 10.9% and 2.2% of patients that underwent laminectomy with high-speed drills and pediatric craniotome drill with a footplate attachment^8^. Moreover, in a study by Romeo et al., different bone cutting methods that included high speed (400 000 rpm) and low speed (20 000–40 000 rpm) drills were evaluated in pig cadaver bones, revealing irregularities in depth and shape of bone drilling, an excess of bone fragments, and low-grade thermal damage^26^. Additionally, a recent case report revealed soft tissue damage at the Sylvian vessels due to an inadvertent movement of the surgeon and wrong handling of the burr direction, since cutting burrs have higher risk of grabbing tissue^27^.

Drilling times with the SSB did not differ significantly from the fluted burr. Subjective evaluation during the surgery suggested that the SSB is more efficient at bone removal than the diamond burr, as it chips bone away in small pieces, whereas the diamond burr tip produces bone dust, observations in line with a previous study using diamond burrs^26^. The bone removal capacity of the SSB was similar to the fluted burr. In theory, the combined safety and bone cutting efficiency of the SSB could enable reduction of procedure duration, however this needs to be further investigated in clinical studies.

Heating of the high-speed drill tip can cause thermal injury of nearby neural structures and other soft tissues^12^. Also the ultrasonic bone curetting has been shown induce high temperatures, and Ota el al. reported coagulative necrosis-like changes in the dura mater, probably due to excessive heat generation, when the ultrasonic bone curette was pressed against the dura in a porcine model^25^. Subjective experience from this study suggested that heating of the hand piece was minimal when using SSB. Although heating was not quantitatively measured or confirmed by objective measurements, it was evident from less discoloration of the bone during procedures that the SSB generated less heat than the diamond burr. The heating of the diamond burr was also demonstrated by occasional formation of smoke, which was not seen with SSB. Our observations of experiencing heating with diamond burrs are in line with previous studies, which reported that temperatures above 100 °C were reached when using diamond or steel-tipped burrs in bone drilling without any kind of cooling method employed; and temperatures above 45 °C (a known threshold for nerve damage by heat^28^) were still observed when insufficient cooling was applied^12^. Naturally, quantitative studies should be carried out to confirm this initial and subjective observation, and whether there would be a need to employ cooling methods with, e.g., CO_2_ coolants^29^ when drilling bone with the SSB. With a fluted burr, heating was not experienced. These initial results do not allow determination of whether the heating was different between SSB and the fluted burr, an observation that also warrants future quantitative investigation.

The SSB cutting speed is adjustable and depends on how much the ring is dislocated from the center of the burr head. The surgeon can adjust the effectiveness according to need at different stages of the surgery, allowing the surgeon to remove bone at a slower rate closer to sensitive tissues, for example. The radial movement of the ring is restricted to limit the cutting speed of the device to a safe level. This significantly reduces the chattering effect (sudden and uncontrollable jumping of the burr while cutting).

Whilst this study does have limitations, as only a small number of sheep were used and a small number of burr holes were drilled by only two neurosurgeons, the evidence suggests that the device works as expected and provides the desired benefits. As this is the first preclinical study demonstrating the safety and effectiveness of the SSB for bone drilling in routine neurosurgery, objective conclusions on the heating and ease of use are lacking in this study. Further quantitative investigation in human trials will be able to determine whether these effects are also substantial, in addition to the lack of occurrence of dura lesion.

Our results in living sheep suggest that SSB is a safe and effective tool for common neurosurgical operations and offers clinically relevant benefits. Further clinical studies are warranted to demonstrate the safety, efficacy, and clinical benefits of the use of SSB in humans, as well as in other surgical fields where bone removal is needed. The technology has the potential to be expanded into a wide range of surgical applications (e.g., dentistry and orthopedics) and new emerging technologies, such as minimally invasive and robotic surgeries.

## Data Availability

The data that support the findings of this study are available from the corresponding author upon reasonable request.

## Abbreviations

ARRIVE: Animal research: reporting of in vivo experiments
k: Dura lesion with Kerrison punch
SEM: Standard error of mean
SSB: Surgify Safety Burr
X: Penetrating dura lesion with high-speed burr
X_m_: Penetrating mucosa lesion with high-speed burr
x: Superficial dura lesion with high-speed burr
